# Distribution and diversity patterns of herpetofauna in the Pantabangan-Carranglan Watershed, Nueva Ecija, Caraballo Mountain Range, Philippines

**DOI:** 10.3897/BDJ.7.e31638

**Published:** 2019-02-14

**Authors:** Paul Henric P. Gojo Cruz, Leticia E. Afuang, Juan Carlos T. Gonzalez, William SM. Gruezo

**Affiliations:** 1 Dept. of Biological Science, Central Luzon State University, Science City of Muñoz, Nueva Ecija, Philippines Dept. of Biological Science, Central Luzon State University Science City of Muñoz, Nueva Ecija Philippines; 2 Animal Biology Division, Institute of Biological Sciences, University of the Philippines Los Baños, Los Baños, Laguna, Philippines Animal Biology Division, Institute of Biological Sciences, University of the Philippines Los Baños Los Baños, Laguna Philippines; 3 Adjunct Faculty, Institute of Graduate Studies, University of the Philippines Los Baños, Los Baños, Laguna, Philippines Adjunct Faculty, Institute of Graduate Studies, University of the Philippines Los Baños Los Baños, Laguna Philippines

**Keywords:** Amphibians, Distribution maps, GIS, Luzon, Philippines, Reptiles

## Abstract

The recent extensive survey conducted in the Pantabangan-Carranglan Watershed, located in the Caraballo Mountain Range during the rainy season (October to November) resulted in the recording of fifty-nine (59) species of amphibians and reptiles (17 frogs, 14 skinks, 3 agamids, 6 gekkonid lizards, 2 varanids and 17 snakes). Out of 59 species, 42 species were Philippine endemics and 25 species are recorded only from the Luzon faunal region. Habitat analysis and mapping showed seven habitat types including lowland dipterocarp forest, grassland, lower montane forest, upper montane forest, pine forest, agricultural areas and riparian habitats.

GIS-based distribution mapping showed that the number of individuals and species are high in forest and associated riparian habitats at mid-elevation (1,000–1,250 m a.s.l.). The distribution pattern in the area is influenced by similarity in microclimatic conditions, availability of resources and niches which species can utilise. Species diversity is centred in mid- to high elevation forest and riparian habitats and in less disturbed areas. Snake diversity is adversely affected by increased disturbance, making them good indicators of the health of an area. The abundance-based Jaccard's Similarity Index showed that connected habitats and elevation gradients have higher species similarity.

## Introduction

The Philippines with its 7,614 islands is home to a diverse herpetofauna with 112 species of amphibians and 361 species of reptiles (Diesmos et al. 2015, Uetz et al. 2018). The number of endemic species found in the Philippines is one of the highest in the world with 84% of recorded amphibians and 66% of recorded reptiles considered as endemic.

Proper management, conservation and preservation of the country's resources depend, to a large extent, on the availability of current, comprehensive and reliable information on the nature, distribution, magnitude and potential of these natural resources for sustainable utilisation (Alcala 1986). Knowledge on distribution patterns of species is important in determining protected areas and providing information on the extent of species distribution. Several studies on terrestrial vertebrate species distribution have already been completed, including works on mammalian elevational distribution (Heaney 2001, Alviola et al. 2011, Rickart et al. 2011) and birds (Vallejo Jr et al. 2009, Peterson et al. 2000). Diesmos et al. 2015 and Leviton et al. 2018 provided distribution maps for species of amphibians and snakes in the Philippines, respectively. In the study of the herpetofaunal distribution in Balesin Island, Polillo, Philippines by Gojo Cruz et al. 2016, it was found out that endemic species tend to inhabit the more intact forest in the centre of the island, while introduced species are more common in or around human structures and forest edges. This highlights the significance of maintaining habitat integrity for species conservation. Spatial distribution of individuals and species within habitats have not yet been extensively documented based on available literature. This recent study aims to determine the spatial distribution of individuals and species in a much larger and more complex area with the goal of providing information on areas of high species concentration which may be used in the formulation of conservation efforts for the herpetofauna of the watershed.

## Materials and Methods

**Survey Areas**

**Figure 1.**
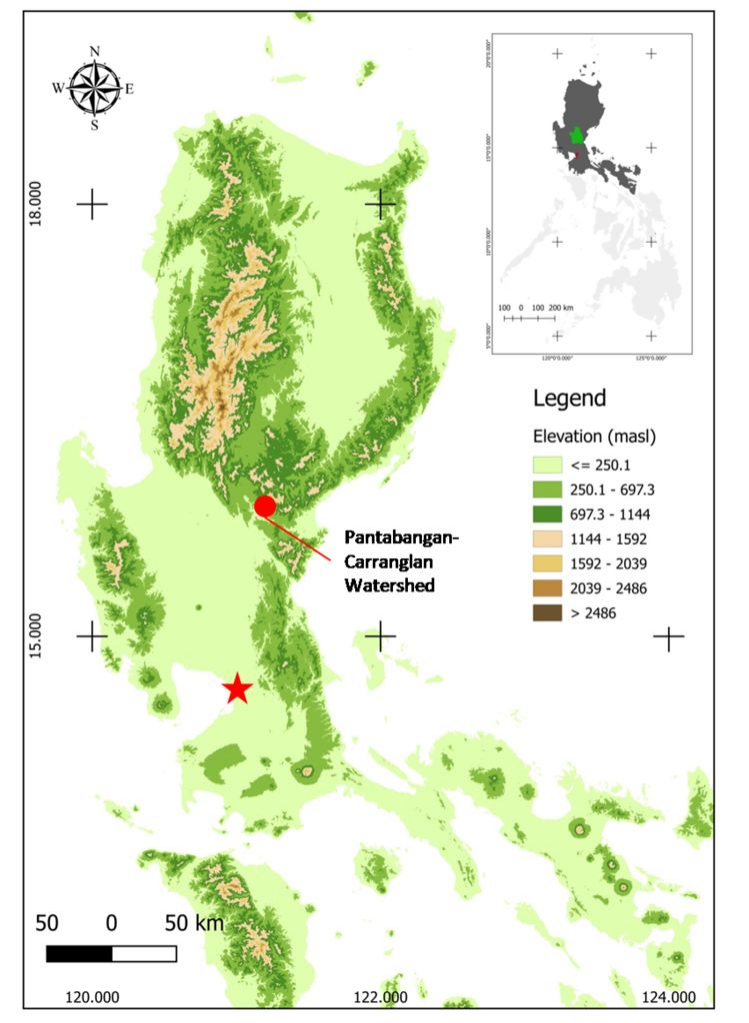
Map of Luzon Island showing the location of the study area. Red star indicates the capital city, Manila.

The Pantabangan-Carranglan Watershed (PCW) lies between 15°44’ and 16°06’ north latitude and 121°00’ and 121°23’ east longitude (WGS 84) (Peras et al. 2008) (Fig. 1). The watershed covers a total area of 97,318 hectares and is part of the Caraballo Mountain Range. Its location falls within Philippine Climate Type I with the rainy season from June to December and with an annual average rainfall of 1,766.5 mm (Lasco et al. 2010, Saplaco et al. 2001). The minimum monthly temperature in the area is 23.21°C and its maximum monthly temperature is 33.71°C (Lasco et al. 2010). The average annual relative humidity recorded in the area is 83.37% with the lowest relative humidity in May (76.6%) and the highest during September (86.67%) (National Power Corporation 1995, National Power Corporation 1997).

Survey areas included the primary forest, pine forest, grassland areas and agricultural plots located near Sitios Calisitan and Binbin, Baranggay Generel Luna in Carranglan, Nueva Ecija. Areas were surveyed using standardised 10 m x 100 m strip transects (Supsup et al. 2016, Diesmos 2008, Diesmos et al. 2005, Roedel and Ernst 2004, Heyer et al. 1994) distributed at varying elevations ranging from 700–1,350 m a.s.l. Sampling was done twice daily (1300–1700 hr and 1900–2300 hr) involving 10 personnel. Data gathering coincided with the rainy season in the area which occurs from August to December. All accessible microhabitats confined within each habitat where animals may be ensconced were searched by raking the forest floor litter, probing epiphytes and tree hollows, upturning rocks and logs and splitting open decayed logs (Diesmos 2008).

Vegetable and rice farming are the primary source of income for the residents in the area. Agricultural plots are located in all accessible sites primarily on flat terrains and sloping sides of the mountains. Grassland areas around plots were cleared to accommodate vegetable plots. The forested areas were left since these areas serve as reservoirs for water used in planting. Other anthropogenic disturbances in the area includes logging, wildlife hunting and small-scale mining. Evidence of logging was still present during the survey, with several finished boards of timber products being seen. Traps for large mammals, such as wild pigs and jungle fowls, were also observed, although the locals said that animals caught were mostly for domestic consumption and that hunting is seasonal only. Traces of small-scale mining was also evident within the forest, but most of the tunnels have already been closed following the strict implementation of anti-mining laws promulgated by the community. An emerging threat to the area's wildlife is the expansion of several game fowl farms in the area which resulted in the clearing of several pine tree stands. Moreover, locals report that the guards of these game fowl farms regularly shoot both domesticated and wild animals to protect the fowl.

Aside from anthropogenic activities, natural calamities, including strong typhoons which result in landslides and increased rate of natural grass fires, are threatening the wildlife of the area.

### Vegetation Mapping

Quantum GIS Nødebo (ver. 2.16.3) (Quantum GIS Development Team 2016) and the latest Google Earth Pro™ Imagery together with the coordinates taken with the GPS were used to plot a vegetation map of the area.

Fernando (2009) recognised 12 forest types in the Philippines based on the physical characteristics of the habitat, vegetation structure and physiognomy. This classification system was used in the description of the habitat types found in the area.

### Collection and Preservation of Voucher Specimens

Voucher specimens for species were collected under Wildlife Gratuitous Permit no. III-2015-06 and preserved using standardised preservation techniques (Heyer et al. 1994). Specimens were euthanised using ethyl acetate. Vouchers were initially fixed in 10% buffered formalin and were eventually stored in 70% alcohol. Collected specimens are deposited at the BioMuseo of the Department of Biological Sciences, CAS, CLSU.

### Distribution Mapping

Individuals encountered were geo-tagged using a hand-held GPS device (Garmin eTrex10). The location of each individual was incorporated in the generated vegetation map through Quantum GIS. Distribution maps were created for aquatic frogs, terrestrial frogs, skinks, agamid and gekkonid lizards and snakes for easy visualisation. A density map showing areas of high individual concentration was also generated using the Heat Map tool in Quantum GIS.

### Diversity Assessment

The Shannon-Wiener function (H’) was computed using the formula: **H’=-∑plnp**, where: p = number of individual per species/total number of individuals; ln = natural logarithm.

The Shannon’s-Wiener function was computed for three categorical elevational gradients in this study: Low (elevations between 700–1,016 m a.s.l.), middle (elevations between 1,017–1,183 m a.s.l.) and high (elevations between 1,184 – 1,350 m a.s.l.). The Diversity Index for the different habitat types was also computed. Habitat types included grassland-pine areas, riparian habitats, lowland dipterocarp forest, lower montane forest and upper montane forest. The diversity between edge and interior habitats and disturbed and undisturbed habitats was also assessed. The generated distribution map aided in the determination of species-area association.

Since the Shannon-Wiener function gives the results in bits per species (Krebs 1999), Hill's number (N_1_) (Hill 1973) was used to convert the value into number of species, using the formula:

N_1_ = e^H'^

where: N_1_ = Hill's number, number of equally common species which would produce the same diversity as H'

e = 2.71828

H' = Shannon-Wiener function

The abundance based Jaccard Index of Similarity (j) (Chao et al. 2006) was used to determine similarities between elevation gradient and habitat type. The formula follows that of : **j= UV/ U + V – UV**, where, U = total relative abundances of the shared species in sample 1; V = total relative abundances of the shared species in sample 2.

The total Relative Abundance was computed using: **Rel. Abundance = Count in sample X/Total Count in sample X**

## Results

### Vegetation Analysis and Mapping

**Figure 2.**
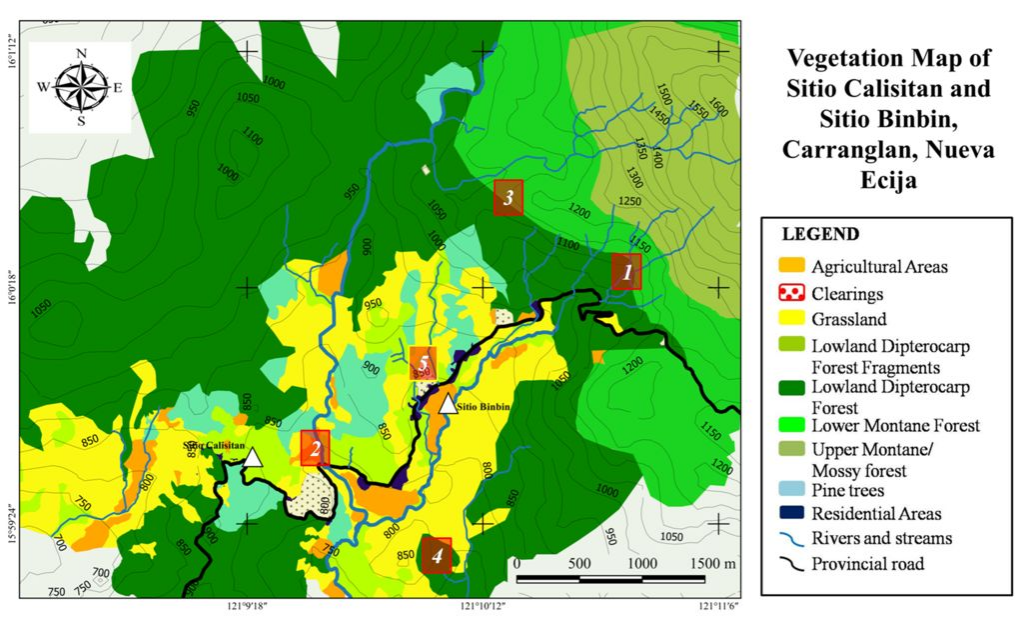
Vegetation map of Sitio Calisitan and Sitio Binbin created using Quantum GIS and the latest Google Earth™ imagery. Numbers in red boxes indicate the study areas.

The survey allowed us to identify seven (7) major habitat types in the area. These includes lowland dipterocarp forest, grassland, lower montane forest, upper montane forest, pine forest, agricultural areas and riparian habitats. The size and extent of each habitat type is shown in Fig. 2. Fifty-nine (59) species of herpetofauna were recorded from the area, of which 6 species were reported by the locals but were not observed during the survey Table 1.

### Species Account

It is surprising that, despite extensive surveys of the riparian, forest habitats and Pandan axils, we did not detect arboreal species of frogs (e.g. *Platymantis sierramadrensis*, *Philautus surdus*, *Rhacophorus pardalis*) which are otherwise recorded in studies from other parts of Luzon (Brown et al. 2000; Fuiten et al. 2011; McLeod et al. 2011; Siler et al. 2011). Our survey method permitted only the survey of the lower strata of the habitat types, primarily the forest floor and lower vegeation, which may explain the low number of arboreal species detected. Species detection was also made difficult by the lack of vocal activities from frogs. Cryptic species, such as burrowing snakes (family Typhlopidae), may also have been overlooked during the survey. Locals report that many large-bodied reptiles such as *Varanus marmoratus*, *Varanus bitatawa*, *Malayopython reticulatus* and *Ophiophagus hannah* are more common during the dry months which fall between March to June. We were unable to detect these species during the survey which coincided with the rainy season. The presence of *O. hannah* was confirmed during the earlier ocular survey of the area by the presence of carcasses killed by farmers. Surveys covering both the dry and wet season are recommended to determine variation in species composition and to gain more reliable information on species abundance, distribution and conservation status which cannot be provided by single site visits (Brown et al. 2013, Diesmos 2008). We also suspect that additional species will eventually be recorded from the area once other parts of the watershed are surveyed and studies conducted in Sierra Madre (Diesmos et al. 2005) and the Cordillera’s (Brown et al. 2012) have shown that herpetological diversity of a general area increases as follow-up visits focusing on different habitat types, forest communities, geological features of the landscape and varying atmospheric conditions are conducted. Follow-up visits are therefore crucial in increasing our knowledge of actual species presence or absence.

### Distribution Patterns of Species

**Figure 3.**
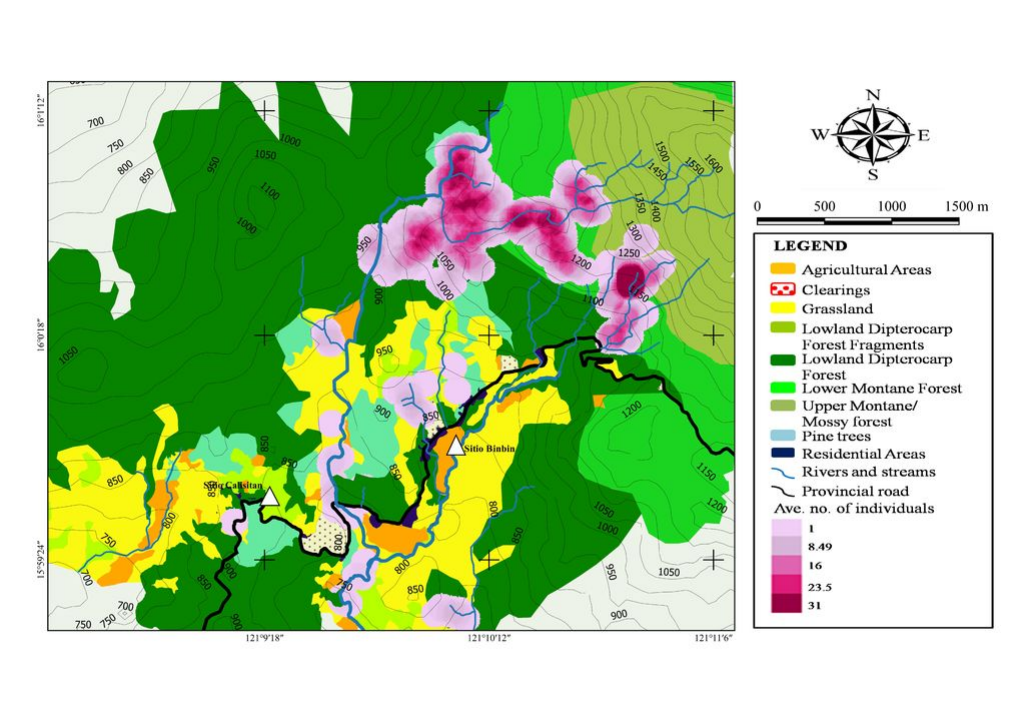
Vegetation map of Sitio Calisitan and Sitio Binbin, General Luna, PCW, Carranglan, Nueva Ecija (2016) showing regions with high concentration of herpetofauna (in purple).

Based on the heatmap index, areas with the highest concentration of individual (dark purple areas) have an average of 31 individuals of herpetofauna (Fig. 3). Species and individual concentrations are particularly high in areas around streams, Binbin River and the forested areas. The presence of ecotones or intermediate habitats forms “edge-effect” (Gonzalez and Dans 1997), i.e. the merging of two habitat types often result in the mixing of herpetofaunal elements from the adjacent habitat types.

Fewer species and individuals were encountered in areas higher than 1,250 m a.s.l. Endemic species were more common in forest areas while native and introduced species were more common in elevations below 900 m a.s.l. around moderately to heavily disturbed areas (i.e. forest fragments, residential and agricultural areas). Native and introduced species were more tolerant and likely benefited from anthropogenic activities, whereas endemic species have lower tolerance to disturbances resulting from human activities.

### Amphibians

The largest concentration of amphibians was found in or around riparian habitats in mid to high elevation forests. Terrestrial species such as *Platymantis* spp., were found farther from water bodies. Delima et al. (2007) and Diesmos (2008) attributes this to differences in reproductive modes, where direct developers (species that do not have a tadpole stage) tend to occur in areas away from water sources while non-direct developers (those with tadpole stage) are concentrated around water bodies. In particular, the spatial distribution of suitable nesting and breeding sites sets limits to the distribution of amphibians (Vitt and Caldwell 2014).

**Figure 4.**
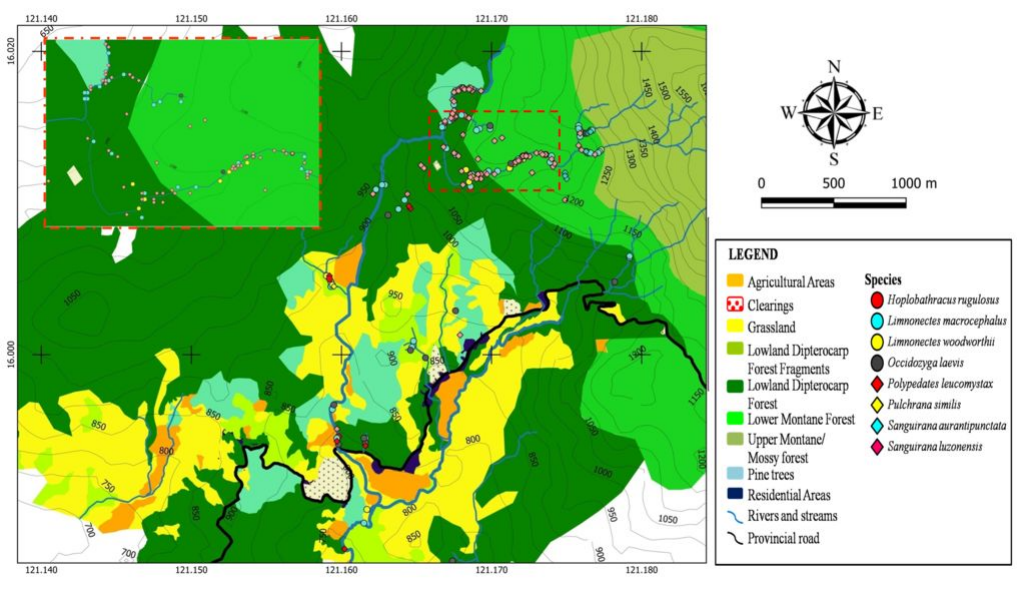
Spatial distribution of aquatic, semi-aquatic and arboreal frogs (Family Dicroglossidae, Ranidae and Rhacophoridae) recorded from Calisitan and Binbin, General Luna, PCW, Carranglan, Nueva Ecija 2016. Inset: Enlarged view of one area of high individual concentration.

A distribution map of aquatic, semi-aquatic and rhacophorid frogs is presented in Fig. 4. *Limnonectes macrocephalus* was found from the lower Binbin River up to large streams at 1,250 m a.s.l. where numerous large individuals (>300 g body weight) were collected. *Hoplobatrachus rugulosus* and *Polypedates leucomystax* were found in rice paddies around Binbin River. It is observed that *Sanguirana luzonensis* and *Sanguirana aurantipunctata* occupy different portions of streams. A dense population of *S. luzonensis* was observed on riparian habitats near the 2^nd^ and 3^rd^ study area (16.0121°N, 121.1680°E and 16.0125°N, 121.1741°E, respectively) while they are rarely found in 1st study area which is characterised by a steep slope with fast-flowing water. This indicates that *S. luzonensis* prefers to inhabit areas with a moderately steep slope and slow water flow. *S. aurantipunctata* becomes more common than *S. luzonensis* in higher elevation streams. This shows niche partitioning between the two species by occupying different portions of streams.

**Figure 5.**
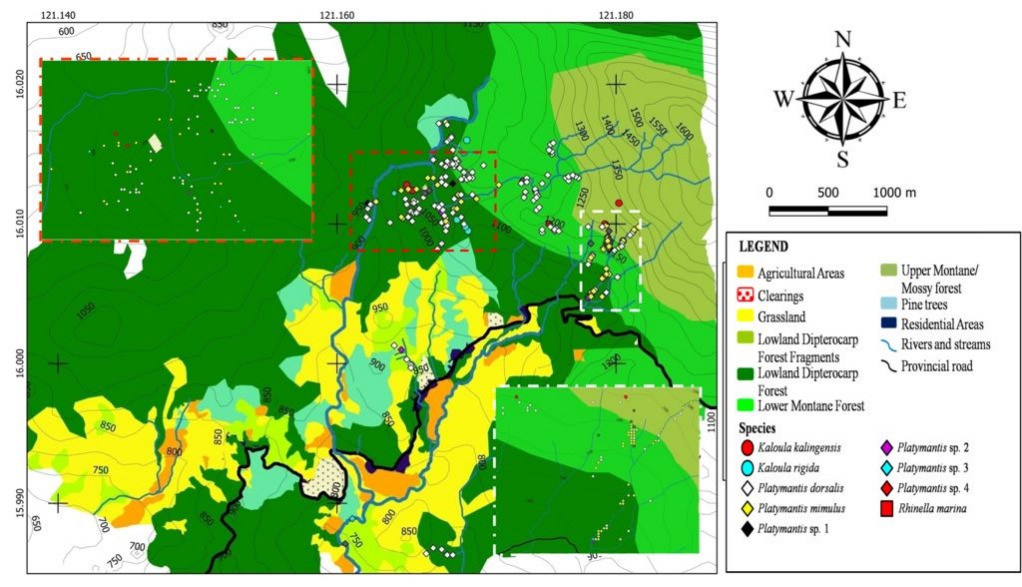
Spatial distribution of terrestrial frogs (Family Bufonidae, Ceratobathrachidae and Microhylidae) recorded from Calisitan and Binbin, General Luna, PCW, Carranglan, Nueva Ecija, 2016. Inset: Enlarged view of areas of high individual concentration.

Spatial distribution of terrestrial frogs is presented in Fig. 5. *Platymantis* spp. were found in varying elevations; however, most were found on mid-elevation dipterocarp forest with a few individuals in forest fragments. *Platymantis* spp. are an important indicator of the relative humid microclimate of the forest (Gonzalez and Dans 1997) thus they can serve as indicator species on the quality of the microhabitat. Density of leaf-litter frogs decreases as elevation increases. Few individuals were collected from elevations greater than 1,300 m a.s.l. Microhylids (*Kaloula kalingensis* and *Kaloula rigida*) were mostly encountered starting from mid-elevation dipterocarp forest up to the lower portions of the montane forest. The invasive *Rhinella marina*, on the other hand, were restricted around residential areas and agricultural plots (<800 m a.s.l.), with a few individuals collected from an upland rice field at 1,050 m a.s.l. These individuals, by hiding in sacks of fertilisers, were likely accidentally brought to the forested areas.

### Reptiles

**Figure 6.**
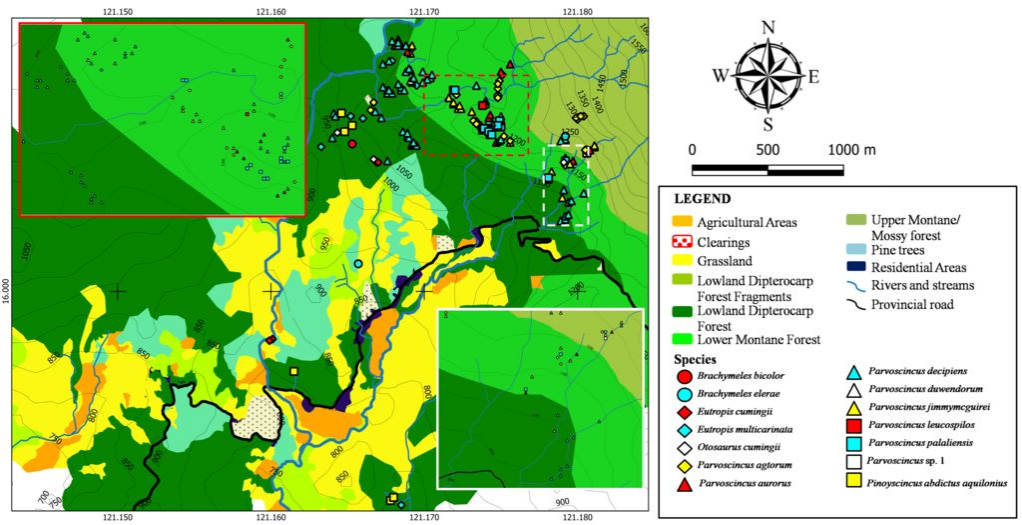
Spatial distribution of skinks (Family Scincidae) recorded from Calisitan and Binbin, General Luna, Carranglan, PCW, Nueva Ecija, 2016. Inset: Enlarged view of areas of high individual concentration.

Fig. 6 shows the spatial distribution of skinks in the area. Members of the genus *Parvoscincus* are widely distributed throughout the forested areas from 950–1,350 m a.s.l. where they are more abundant compared to large-bodied skinks such as *Eutropis cumingi, Eutropis multicarinata borealis* and *Otosaurus cumingi.* These large-bodied skinks are more abundant from lower areas up to the middle portion of the mountain. Fossorial skinks (*Brachymeles elerae* and *B. bicolor*) were observed in the forest up to the mossy forest. *B. bicolor, O. cumingi* and *Parvoscincus leucospilos* showed major elevational range extensions. Siler et al. (2014) reported that *P. leucospilos* is common at elevations from 200–800 m a.s.l.; our specimen was collected at 1,150 m a.s.l. The upper elevation limit for *B. bicolor* was 850 m a.s.l. (Brown and Duya 2009) and for *O. cumingi*, it was 1,000 m a.s.l. (Brown and Rico 2009). The single *B. bicolor* from our study area was collected at 980 m a.s.l. One of the *O. cumingi* we recorded was observed at 1,170 m a.s.l.

**Figure 7.**
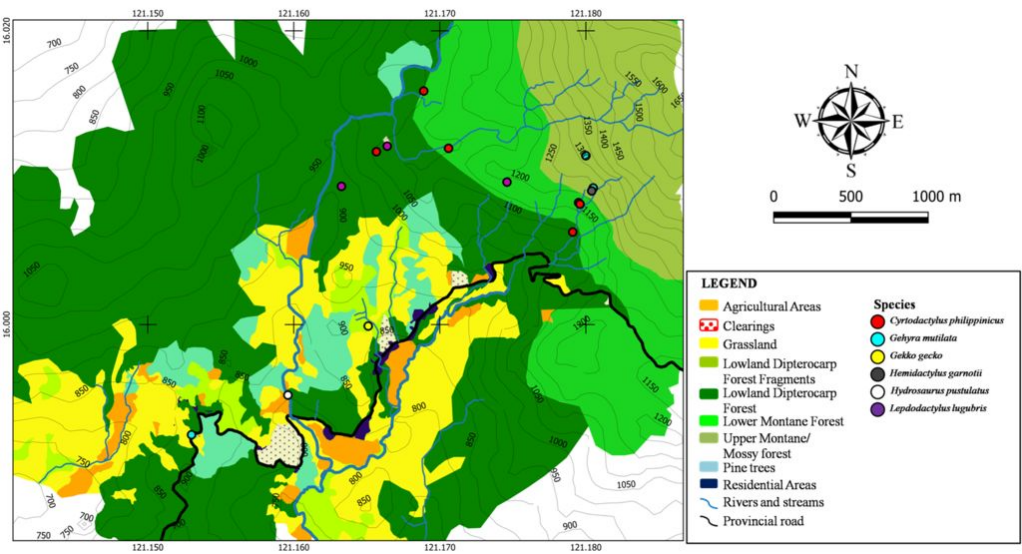
Spatial distribution of gekkonid and agamid lizards (Family Gekkonidae and Agamidae) recorded from Calisitan and Binbin, General Luna, PCW, Carranglan, Nueva Ecija, 2016.

Gekkonid and agamid species were sparsely distributed throughout Calisitan and Binbin, PCW, Carranglan (Fig. 7). Few species and individuals of gekkonid and agamid lizards were recorded from the area attributed to the limited sampling of the canopy. The method employed was limited to surveying of the lower strata of the forest. Temporal variations in species activity also influence the detection of species. Locals report that varanids and some agamids are more common during the dry months than in the wet months. This might explain the low encounter rates for large-bodied lizards. Human commensals such as *Gehyra mutilata* and *Hemidactylus frenatus* are often found in human habitation or in lowland dipterocarp forest where they are found inhabiting sheds built by farmers. Lepidodactylus cf. lugubris, on the other hand, is strictly found in the middle portions of forest where they are commonly encountered in areas with a more or less open-canopy. Due to the few individuals of agamids and varanids observed during the survey, the distribution patterns of these taxa cannot be assessed.

**Figure 8.**
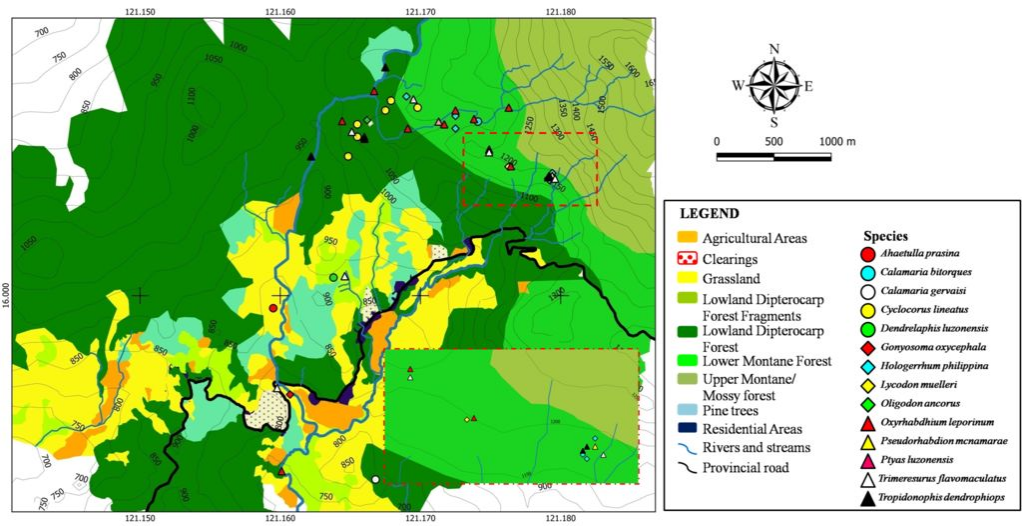
Spatial distribution of snakes recorded from Calisitan and Binbin, General Luna, Carranglan, Nueva Ecija, 2016. Inset: Zoomed in view of one area of high individual concentration.

Fig. 8 shows the spatial distribution of snakes recorded from the area. *Gonyosoma oxycephalum, Dendrelaphis luzonensis* and *Ahaetulla prasina preocularis* were found in lower areas, while the other snake species were limited to the forest. *Trimeresurus flavomaculatus, Hologerrhum philippinum, Oxyrhabdium leporinum* and *Tropidonophis dendrophiops* were recorded in the different areas of the forest. We were unable to detect the presence of *Cyclocorus lineatus* in the 1^st^ study area (16.0092°N, 121.1805°E) despite extensive surveys. The intervening forest characteried by rugged terrain between the 1^st^ and the 2^nd^ study area may have prevented this species from reaching the south-eastern parts of the mountain. The distribution of snakes is influenced both by the physical structure of the microhabitat and the availability of prey items. The high number of snakes found around riparian habitats is attributed to the presence of numerous frogs which constitutes a large part of the snakes’ diet.

Elevational range extension was detected for *Calamaria bitorques* (1,143 m a.s.l.), *Lycodon muelleri* (1,235 m a.s.l.), *Oligodon ancorus* (1,065 m a.s.l.), *Oxyrhabdium leporinum* (1,245 m a.s.l.) and *Trimeresurus flavomaculatus* (1,201 m a.s.l.). According to the data, the upper elevation limitations are *C. bitorques* 850 m a.s.l.(Brown et al. 2009a), *O. ancorus* (600 m a.s.l.) (Brown et al. 2009b), *O. leporinum* (1,100 m a.s.l.) (Brown and Rico 2009) and *T. flavomaculatus* (700 m a.s.l.) (Brown et al. 2009c). Brown et al. (2013) reported that *L. muelleri* is commonly found at elevations lower than 500 m a.s.l. Interspecific and intraspecific competition may be reduced if more elevational gradients and areas are utilised by species.

### Diversity Patterns

Table 2 presents the computed Shannon-Wiener function (H’) and Hill's number (N_1_) for the recorded herpetofauna in the study area.

### Herpetofaunal diversity at different elevational gradients

As elevation increases, the number of individuals and levels of diversity decreases amongst lizards and snakes. Hill's number for frogs at higher parts of the mountain (>1,184 m a.s.l.) is higher compared to the computed value for frogs at the middle portion (1,017–1,183 m a.s.l.). Since the Shannon-Wiener function takes into account species richness and abundance, this resulted in the lower computed value of H’ for frogs at mid-elevation which is also reflected by Hill's number (6.17 species vs. 8.85 species). The area is less heterogenous, 65% of all the frogs recorded in that elevation gradient is represented by four species (*Platymantis dorsalis, P. mimulus, Sanguirana luzonensis* and *Limnonectes macrocephalus*). Diversity and abundance of lizards and snakes are negatively affected by elevation. The effect of elevation on temperature and humidity affecting egg development and thermal physiology may be important, especially for reptiles (Navas 2002, Delima et al. 2007). Microclimatic conditions and the presence of key microhabitats are also important prime ecological correlates influencing the herpetofaunal community (Scheffers et al. 2013, Diesmos 2008). The observed pattern is consistent with those observed in Mt. Hamiguitan (Delima et al. 2007) and Mt. Makiling (Gonzalez and Dans 1997). According to Diesmos (2008), differences in microclimatic conditions, precipitation and microhabitat structure at different elevation affects the herpetofaunal community. Elevation had a negative effect on abundance of individuals but not herpetofaunal species richness according to Scott (1976). This may be due to the capability of species to tolerate slight changes in elevational microclimatic conditions but with most individuals preferring lower elevations due to more favourable conditions.

Using abundance-based Jaccard’s Similarity Index (Chao et al. 2006), it was shown that middle part and higher elevation habitats shared a number of species (Jaccard’s Similarity Index = 0.85). This similarity could be attributed to shared microclimatic conditions and habitat continuity, allowing individuals to move into adjacent areas at higher or lower elevations. It appears that the middle portion of the mountain serves as the upper limit for some of the species found in the lower parts of the mountain (e.g. *Polypedates leucomystax* and *Gehyra mutilata*) and the lower limit for some of the higher elevation species (e.g. *Brachymeles elerae* and *Hologerrhum philippinum*).

### Herpetofaunal diversity of different habitat types

Species diversity is lowest in grassland-pine habitats (N_1_ = 7.92) while riparian habitats recorded the highest species diversity for all the taxa (N_1_ = 12.94). Amphibians in the grassland-pine area were represented by both native (*Occidozyga laevis* and *Polypedates leucomystax*) and introduced species (*Rhinella marina* and *Hoplobatrachus rugulosus*).

Riparian habitats harboured both native and several endemic species (*Limnonectes* spp., *Sanguirana luzonensis* and *S. aurantipunctata*). Aquatic habitats provide suitable moisture and sites for egg deposition for many species of frogs. The presence of a large number of prey items (frogs and insects) in riparian habitats in turn attracts predators such as snakes and lizards. Riparian vegetation is important in maintaining low water temperatures and also increases connectivity of different habitat types (Gomez-Roxas et al. 2005).

In Mt. Makiling, diversity of amphibians and reptiles is high in mid-montane dipterocarp forest (500–600 m a.s.l.) (Gonzalez and Dans 1997) which is different from our observation based on the computed Shannon’s Diversity Index. The decrease in the diversity of frogs and lizards in the dipterocarp forest and lower montane forest in our study area is due to less heterogeneity. *Platymantis dorsalis* and *P. mimulus* accounted for more than 56% of all the recorded frogs in the dipterocarp forest and 72% of the species in the lower montane forest. *Parvoscincus decipiens* accounted for 62% of the recorded lizards in the dipterocarp forest. The Philippine clades of *Platymantis*, *Limnonectes* and *Parvoscincus* (= *Sphenomorphus* s.l.) exhibit a marked prevalence for cryptic speciation (Diesmos 2008), thus misidentification could have affected our computations and resulted in underestimation of the actual diversity in these habitats.

The diversity of reptiles in the upper montane forest declined markedly, with no snakes recorded from this habitat type. It should be noted that the pattern of species diversity based on habitat type is comparable to that observed in the effects of elevation gradient. The decrease in diversity in mossy forest is attributed to less stratification resulting in less habitat variety and available niches (Gonzalez and Dans 1997).

The Similarity Index for the different habitat types showed that riparian habitats have high similarity with dipterocarp forest (0.76), lower montane forest (0.74) and grassland-pine habitat (0.63). This is not surprising since riparian habitats traverse all the other habitat types, making it an important link between habitat types.

### Edge effect on herpetofaunal diversity

Frogs showed a higher diversity at edge habitats (N_1_ = 8.00) , particularly in riparian habitats compared to interior habitats (N_1_ = 5.26). Based on the distribution map, frogs were more common in riparian habitats associated with forest areas compared to those whose banks were covered with less vegetation (e.g. Binbin River).

Lizard diversity was also different between edge and interior habitats, with more species in interior habitats (20 species) compared to edge habitats (12 species). Only snake diversity showed a decline in edge habitats. Diesmos (2008) reported that snakes are more abundant in contiguous forest, with small to medium-bodied species generally fewer in forest fragments. Results of diversity assessment undertaken in Mt. Makiling (Gonzalez and Dans 1997) showed that there is an increase in species diversity from more open to more closed vegetation. Increasing edge effects brought by fragmentation may have a negative effect on snakes found in the area. The high number of individuals and species in forest interiors was also observed in Balesin Island in the Polillo Island, Quezon, Philippines (Gojo Cruz et al. 2016). Most of the native and endemic species in the island were recorded on the intact forest in the centre of the island, attributed to the presence of more microhabitats and sufficient vegetation cover. Our study also showed that increasing edge effects brought by fragmentation may have a negative effect on snakes found in the area since most snakes were recorded within forest interiors.

Changes in forest types result in changes in canopy height and density, often resulting in plant growth and density (Murcia 1995) affecting the available microhabitats. A survey on studies on the effects of habitat change on herpetofauna by Gardner et al. (2007) showed that most studies relating to the importance of edge effect on amphibians and reptiles indicated that edges either have no effect, weak effect or species-specific effect with no over-all change in species richness. Our results showed that the different taxa had different responses to edge effect.

### Effect of disturbance on herpetofaunal diversity

Disturbance in the area includes anthropogenic disturbances in the form of kaingin and logging activities. Natural disturbances include typhoons, landslide and forest fires. Species diversity for all taxa around disturbed sites (around logging areas, agricultural areas and landslide areas) was lower compared to species diversity in less disturbed sites. Diversity of amphibians and reptiles has an inversely proportional relationship to human encroachment in the environment (Gonzalez and Dans 1997).

The computed Shannon-Weiner function and Hill's number values may actually be an underestimation of actual diversity due to the following reasons: (1) cryptic speciation amongst *Platymantis* and *Parvoscincus* spp. which may have resulted in the misidentification of species; (2) limited sampling of canopy habitats; (3) limited sampling area; and (4) six species were excluded from analysis since no individuals were collected during the survey.

High similarity values between adjacent elevation gradients and habitat types showed that distribution patterns of the species are influenced by shared similarity in microclimatic conditions and availability of resources. It was shown that snake diversity was centred on interior habitats and in undisturbed areas. The presence of snakes may be a good indicator of habitat quality based on their preference for undisturbed areas.

## Conclusions

Distribution of herpetofauna in the area is influenced primarily by the microclimatic conditions and availability of resources which a species can utilise. Distribution maps showed that species and individual concentrations are greater in complex habitat types such as forest and riparian habitats compared to less complex habitat types (grasslands, pine forest and agricultural areas). Diversity in the area is centred around mid- to high elevation forests and riparian habitats and in less disturbed, interior areas. Since high species diversity and abundance are concentrated on the riparian habitats and forest areas, conservation of these areas is important. Distribution maps prove to be an important aid for conservation by allowing areas occupied by species to be determined.

## Figures and Tables

**Table 1.** Amphibians and reptiles of Sitio Calisitan and Sitio Binbin, Barangay General Luna, PCW, Carranglan, Nueva Ecija, Caraballo Mountain Range, 2016.

**SPECIES**	**Elevational Range (m a.s.l.)**	**HABITAT TYPE**				
		**Grassland**- **Pine Habitat**	**Riparian Habitat**	**Dipterocarp Forest**	**Lower Montane Forest**	**Upper Montane Forest**
** Amphibia **						
** Bufonidae **						
*Rhinella marina* (Linnaeus, 1758)	800–900		X	X		
** Ceratobatrachidae **						
*Platymantis dorsalis* (Dumeril, 1853)	700–1,300	X	X	X	X	X
*Platymantis mimulus* Brown, Alcala & Diesmos,1999	1,000–1,200		X	X	X	
*Platymantis* sp. 1	950–1,350		X	X	X	X
*Platymantis* sp. 2	1,000–1,250	X	X	X	X	
*Platymantis* sp. 3	1,050–1,200		X	X		X
*Platymantis* sp. 4	1,050–1,100		X	X	X	X
** Dicroglossidae **						
*Hoplobatracus rugulosus* (Wiegmann, 1854)	800–1,000		X			
*Limnonectes macrocephalus* (Inger, 1954)	800–1,250	X	X	X	X	
*Limnonectes woodworthi* (Taylor, 1923)	800–1,250	X	X	X		
*Occidozyga laevis* (Günther, 1858)	750–1,200	X	X	X		
** Microhylidae **						
*Kaloula kalingensis* Taylor, 1922	1,000–1,350			X		X
*Kaloula rigida* Taylor, 1922	1,100–1,250			X	X	X
** Ranidae **						
*Pulchrana similis* (Günther, 1873)	850–950		X			
*Sanguirana luzonensis* (Boulenger, 1896)	850–1,250	X	X	X	X	
*Sanguirana aurantipunctata* Fuiten, Diesmos, Welton, Barley, Oberheide, Rico & Brown, 2011	1,150–1,250		X		X	
** Rhacophoridae **						
*Polypedates leucomystax* (Gravenhorst,1829)	750–950		X	X		
**Reptilia (Lizards)**						
** Agamidae **						
*Draco spilopterus* (Weigmann, 1834)	900–1,000			X		
*Hydrosaurus pustulatus* (Eschcholtz,1829)	800–900		X			
** Gekkonidae **						
*Cyrtodactylus philippinicus* (Steindacher, 1867)	1,050–1,200		X	X	X	
*Gehyra mutilata* (Weigmann, 1834)	750–900	X	X	X		
*Gekko gecko* (Linnaeus, 1758)	850–900		X			
*Hemidactylus frenatus* (Dumeril & Bibron, 1836)	750–900	X				
*Hemidactylus garnoti* (Dumeril &Bibron, 1836)	1,200–1,250			X		
Lepidodactylus cf. lugubris (Dumeril & Bibron, 1836)	900–1,200			X	X	
** Scincidae **						
*Brachymeles bicolor* (Gray, 1845)	950–1,000			X		
*Brachymeles elerae* Taylor, 1917	1,150–1,250	X		X	X	
*Eutropis cumingi* (Brown & Alcala, 1980)	800–1,000		X	X		
*Eutropis multicarinata borealis* (Brown & Alcala, 1980)	750–1,100			X	X	
*Otosaurus cumingi* Gray, 1845	950–1,200			X	X	
*Parvoscincus aurorus* Linkem & Brown, 2013	1,000–1,250		X	X	X	
*Parvoscincus agtorum* Linkem & Brown, 2013	1,050–1,350		X	X	X	X
*Parvoscincus decipiens* (Linkem & Brown, 2013)	950–1,300		X	X	X	
*Parvoscincus duwendorum* Siler, Linkem, Cobb, Watters, Cummings, Diesmos, & Brown, 2014	1,100–1,200				X	
*Parvoscincus jimmymcguirei* Linkem & Brown, 2013	1,100–1,250		X		X	X
*Parvoscincus leucospilos* (Peters, 1872)	1,150–1,200				X	
*Parvoscincus palaliensis* Linkem and Brown, 2013	1,100–1,200		X		X	
*Parvoscincus* sp.	1,200–1,225		X			
*Pinoyscincus abdictus aquilonius* (Brown & Alcala, 1980)	750–1,000		X	X		
**Reptilia (Snakes)**						
** Colubridae **						
*Ahaetulla prasina preocularis* (Taylor, 1922)	800–850		X			
*Calamaria bitorques* Peters, 1872	1,100–1,150		X			
*Calamaria gervaisi* Dumeril & Bibron,1854	750–800			X		
*Dendrelaphis luzonensis* Leviton, 1961	850–900	X				
*Gonyosoma oxycephalum* (Boie, 1827)	750–800	X				
*Lycodon muelleri* Dumeril, Bibron & Dumeril,1854	1,150–1,200				X	
*Oligodon ancorus* (Girard, 1858)	1,050–1,100			X		
Pseudorhabdion cf. mcnamarae (Taylor, 1917)	1,150–1,200		X			
*Ptyas luzonensis* (Günther, 1873)	1,100–1,150		X			
*Tropidonophis dendrophiops* (Günther, 1883)	900–1,200		X	X	X	
**Lamprophiiidae**						
*Cyclocorus lineatus lineatus* (Reinhardt, 1843)	1,000–1,100			X	X	
*Hologerrhum philippinum* Günther, 1858	1,050–1,150				X	
*Oxyrhabdium leporinum leporinum* (Günther, 1858)	750–1,200	X	X	X	X	
** Viperidae **						
*Trimeresurus flavomaculatus* (Gray, 1842)	800–1,200	X	X	X		
**TOTAL**		**13**	**34**	**34**	**28**	**8**

**Table 2.** Diversity patterns for the herpetofauna collected in Sitios Calisitan and Binbin, PCW, Carranglan, Nueva Ecija, 2016. Numbers in parenthesis indicated the computed Hill's number (N_1_) given as number of species. Descriptive classification of the Shannon-Weiner function follows Fernando (1998).

**PARAMETER**	**TAXA**			**OVER-ALL SPECIES DIVERSITY**
	**Frogs**	**Lizards**	**Snakes**	
**Elevation Gradient**				
Low	2.19 (8.94)	2.26 (9.58)	1.97 (7.17)	2.97 (19.49) = Moderate
Middle	1.82 (6.17)	1.86 (6.42)	1.87 (6.49)	2.59 (13.33) = Moderate
High	2.18 (8.85)	1.78 (5.93)	1.56 (4.76)	2.71 (15.03) = Moderate
**Habitat type**				
Grassland-Pine Habitat	1.52 (4.57)	0.69 (1.99)	1.39 (4.01)	2.07 (7.92) = Low
Riparian Habitat	1.98 (7.24)	2.24 (9.39)	1.56 (4.76)	2.56 (12.94) = Moderate
Lowland Dipterocarp Forest	1.74 (5.70)	1.47 (4.35)	1.54 (4.66)	2.39 (10.91) = Low
Lower Montane Forest	1.33 (3.78)	1.94 (6.96)	1.57 (4.81)	2.43 (11.36) = Low
Upper Montane Forest	2.01 (7.46)	1.15 (3.16)	0 (1.00)	2.16 (8.67) = Low
**Effect of edge**				
Interior	1.66 (5.26)	2.13 (8.41)	1.87 (6.49)	2.59 (13.33) = Moderate
Edge	2.08 (8.00)	2.17 (8.76)	1.81 (6.11)	2.7 (14.88) = Moderate
**Effect of Disturbance**				
Undisturbed Areas	1.95 (7.03)	2.11 (8.25)	2.11 (8.25)	2.73 (15.33) = Moderate
Disturbed Areas	1.95 (7.03)	2.11 (8.25)	1.33 (3.78)	2.48 (11.94) = Low
